# A Novel Risk-Score Model With Eight MiRNA Signatures for Overall Survival of Patients With Lung Adenocarcinoma

**DOI:** 10.3389/fgene.2021.741112

**Published:** 2021-11-12

**Authors:** Jun Wu, Yuqing Lou, Yi-Min Ma, Jun Xu, Tieliu Shi

**Affiliations:** ^1^ Center for Bioinformatics and Computational Biology, And the Institute of Biomedical Sciences, School of Life Sciences, East China Normal University, Shanghai, China; ^2^ Department of Pulmonary Medicine, Shanghai Chest Hospital, Shanghai Jiao Tong University, Shanghai, China; ^3^ Department of Emergency Medicine, The First Hospital of Anhui Medical University, Hefei, China; ^4^ Beijing Advanced Innovation Center for Big Data-Based Precision Medicine, Beihang University and Capital Medical University, Beijing, China

**Keywords:** lung adenocarcinoma, microRNA signature, risk-score model, overall survival time, treatment response

## Abstract

Lung adenocarcinoma (LUAD) is the most common subtype of lung cancer with heterogeneous outcomes and diverse therapeutic responses. To classify patients into different groups and facilitate the suitable therapeutic strategy, we first selected eight microRNA (miRNA) signatures in The Cancer Genome Atlas (TCGA)-LUAD cohort based on multi-strategy combination, including differential expression analysis, regulatory relationship, univariate survival analysis, importance clustering, and multivariate combinations analysis. Using the eight miRNA signatures, we further built novel risk scores based on the predefined cutoff and beta coefficients and divided the patients into high-risk and low-risk groups with significantly different overall survival time (*p*-value < 2 e−16). The risk-score model was confirmed with an independent dataset (*p*-value = 4.71 e−4). We also observed that the risk scores of early-stage patients were significantly lower than those of late-stage patients. Moreover, our model can also provide new insights into the current clinical staging system and can be regarded as an alternative system for patient stratification. This model unified the variable value as the beta coefficient facilitating the integration of biomarkers obtained from different omics data.

## Introduction

Lung cancer, which is one of the most common and severe types of cancer, remains the leading cause of cancer incidence and mortality worldwide in both males and females (Siegel et al., 2019). Lung adenocarcinoma (LUAD) is the most prevalent histological subtype of lung cancer, with an increasing incidence over the past few decades (Ferlay et al., 2010). The traditional clinical staging system for LUAD, which is based on anatomical information, appears to be inadequate for prognosis evaluation or treatment choices now due to the heterogeneity among patients.

With the rapid advance of molecular biology, many diagnostic and prognostic biomarkers have been identified for various cancers (Wang et al., 2017a; Wang et al., 2017b; Cheng et al., 2019; Huang et al., 2020a; Sheng et al., 2020). With the use of these biomarkers, the traditional tumor classes can be further divided into new subtypes, which may benefit from different therapeutic strategies (Li et al., 2019; Sherafatian and Arjmand, 2019; Lathwal et al., 2020). Besides that, most targeted agents (e.g., cetuximab, gefitinib, and tamoxifen) are effectively only if their respective targets are mutated or differentially expressed (Sun et al., 2017; Yang et al., 2020).

MicroRNAs (miRNAs) are small non-protein-coding RNAs, which can negatively regulate gene expression by binding to their selective messenger RNAs (mRNAs), thereby influencing various biological progresses, such as cellular differentiation, cell-cycle control, and apoptosis (Bentwich, 2005; Cheng et al., 2005; Novello et al., 2013). MiRNAs are reported to be differentially expressed in various human cancers and act as both tumor suppressors and oncogenes (Volinia et al., 2006; Cui et al., 2020). For some certain types of cancer, the miRNAs are proved to be more effective in cancer classification than mRNAs (Miska, 2007), and the miRNAs are also used as signatures for prognosis prediction. Yu et al. identified five miRNAs significantly associated with patient relapse and survival based on 117 non-small cell lung cancer (NSCLC) patients (Yu et al., 2008). Li et al. also identified eight miRNAs as signatures for survival prediction in LUAD (Li et al., 2014). Similarly, Hess et al. provided a five-miRNA signature, which is a strong and independent prognostic factor for disease recurrence and survival of patients with HPV-negative head and neck squamous cell carcinoma (HNSCC) (Hess et al., 2019). All these results showed that miRNAs are powerful potential signatures for prognosis prediction. However, there were very few overlaps between these miRNA signatures identified by different groups. Moreover, most studies just focused on the miRNA or mRNA expression level independently and ignored the negatively regulative relationship between miRNAs and mRNAs.

In this study, based on the miRNA expression, gene expression profiles and clinical information of 516 LUAD samples from The Cancer Genome Atlas (TCGA) (The Cancer Genome Atlas Research Network, 2014), we built the miRNA–gene negative regulation pairs to ensure that the candidate miRNAs influence biological progress of these samples. Then, we screened eight miRNA signatures through differential expression analysis, regulatory relationship filtering, univariate survival analysis, importance clustering, and multivariate combination selection. Based on the eight miRNA signatures, we built a risk-score model to group the patients as high-risk and low-risk. The model performance was further proved using an independent dataset. We demonstrated that the model can also be used for stratification of patients in the same tumor stage.

## Results

### Data Collection

The gene expression, miRNA expression, and clinical data of TCGA-LUAD were download from UCSC Xena (http://xena.ucsc.edu) (Goldman et al., 2017). Besides that, we also downloaded the miRNA expression and related clinical data of LUAD from the Clinical Proteomic Tumor Analysis Consortium (CPTAC)-3 database (Edwards et al., 2015) using the R/Biconductor package “TCGAbiolinks” as the independent validation data (Colaprico et al., 2016; Mounir et al., 2019). Only the primary solid tumor (TP) and solid tissue normal (NT) samples were selected. Patients with less than 30  days of overall survival (OS) were excluded to avoid the possible unrelated causes of death. The details of the samples are shown in Table 1.

**TABLE 1:** Number of samples obtained from different databases.

TCGA-LUAD				CPTAC-LUAD	
Gene expression		MiRNA expression		MiRNA expression	
TP	NT	TP	NT	TP	NT
510	58	510	45	111	102

Note. OS, overall survival; TCGA, The Cancer Genome Atlas; LUAD, lung adenocarcinoma; CPTAC, Clinical Proteomic Tumor Analysis Consortium; miRNA, microRNA; TP, primary solid tumor; NT, solid tissue normal.

**FIGURE 1:**
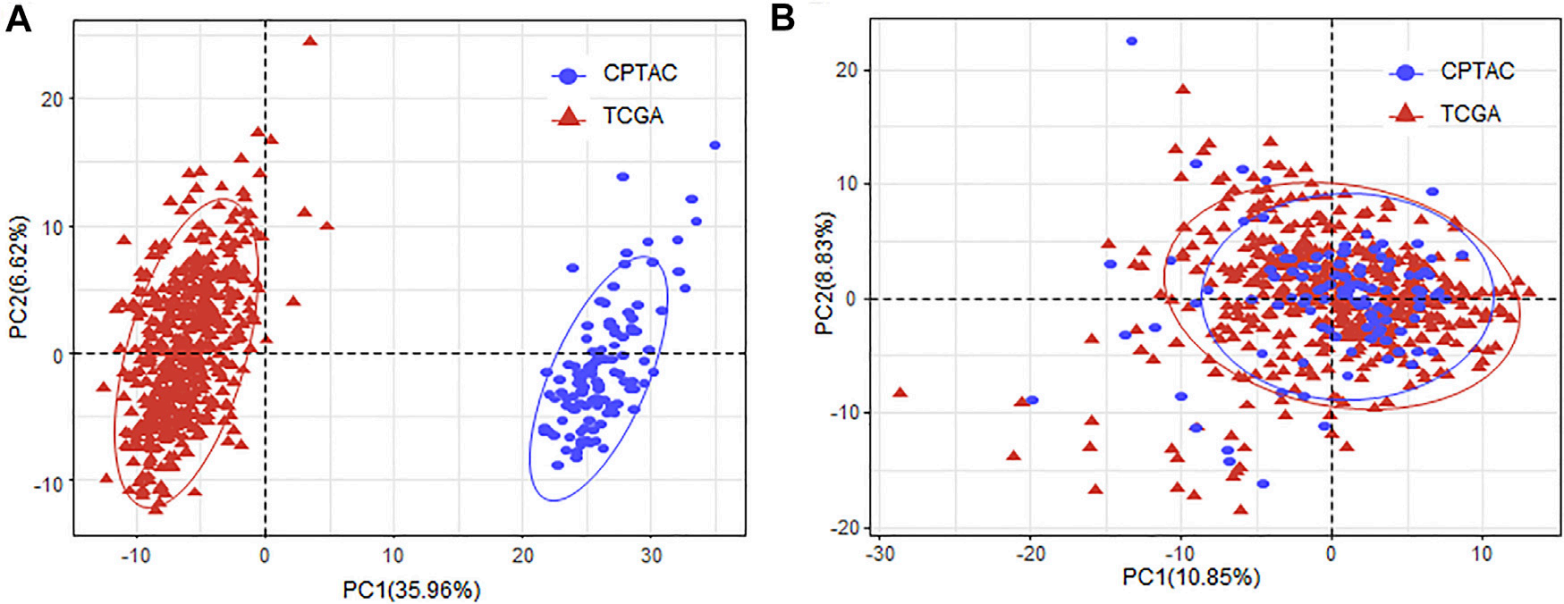
Removing batch effect of the miRNA expression between TCGA and CPTAC datasets. **(A)** PCA plot of the samples obtained from TCGA and CPTAC database with the miRNA expression before batch effect removal. **(B)** PCA plot of the samples obtained from TCGA and CPTAC database with the miRNA expression after batch effect removal. MiRNA, microRNA; TCGA, The Cancer Genome Atlas; CPTAC, Clinical Proteomic Tumor Analysis Consortium; PCA, principal component analysis.

As the miRNA expression was obtained from different databases, we applied ComBat (Leek et al., 2012) to remove the batch effect (Figures 1A,B).

### Differential Gene Expression Analysis

**FIGURE 2:**
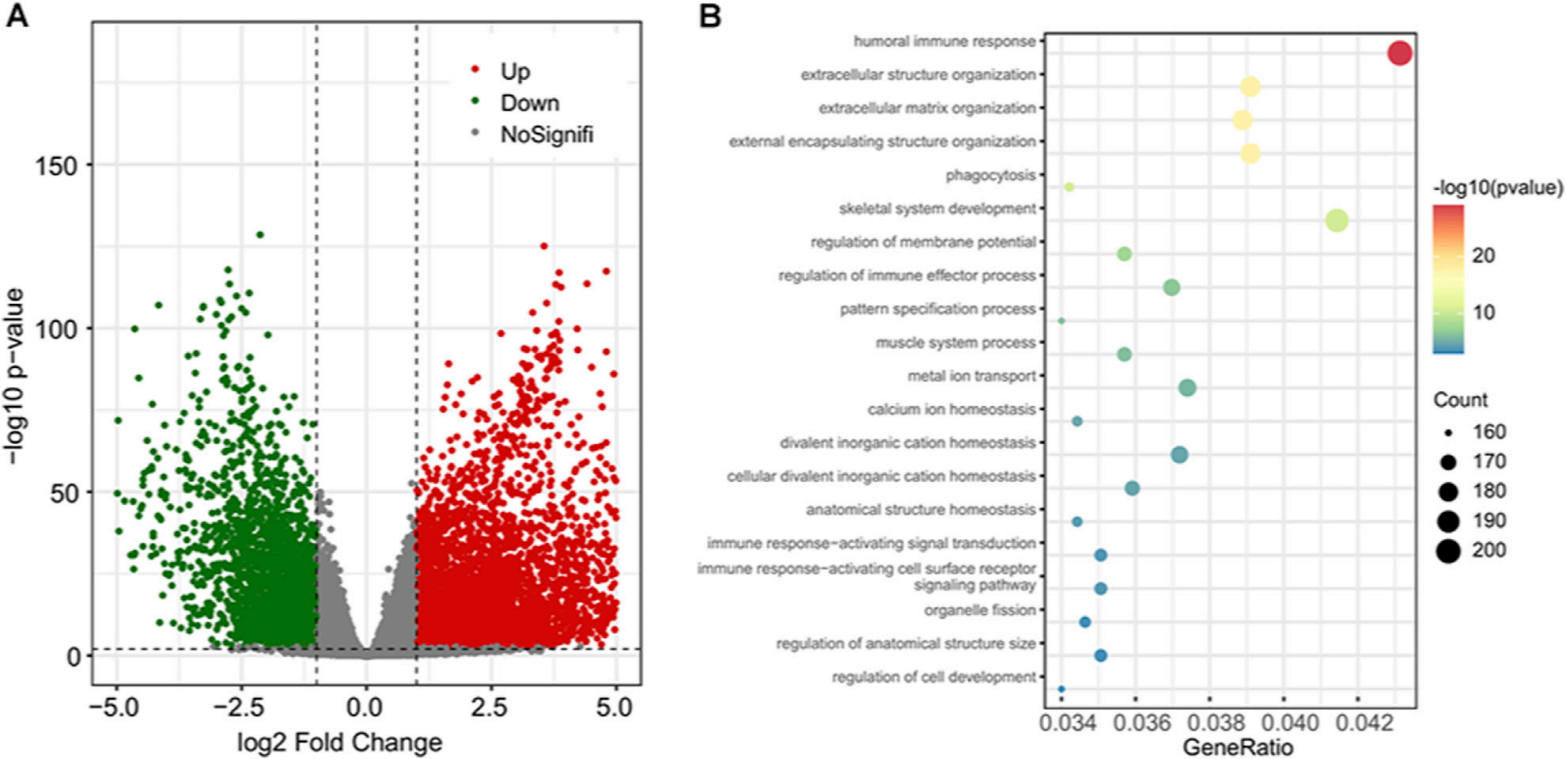
The differential gene expression analysis results and enriched functional terms. **(A)** The volcano plot of the DEGs; 4,522 upregulated genes are in red and 2,531 downregulated genes are in green. **(B)** Bubble plot of the top 20 enriched biological processes. DEGs, differentially expressed genes.

The count data of gene expression were used to perform the differential expression analysis. The genes with adjusted *p*-value of less than 1 e−3 and absolute log2 fold change ≥1 were regarded as significantly differentially expressed. As a result, a total of 4,522 (64.11%) upregulated and 2,531 (35.89%) downregulated genes (Figure 2A). The Gene Ontology (GO) term and Kyoto Encyclopedia of Genes and Genomes (KEGG) pathway enrichment analysis results showed that these differentially expressed genes (DEGs) were enriched in 842 biological processes (BPs), 161 molecular functions (MFs), 137 cellular components (CCs), and 44 KEGG pathways (Figure 2B; Supplement Table S1).

### MicroRNA Signature Identification Based on Multi-Strategy

**FIGURE 3:**
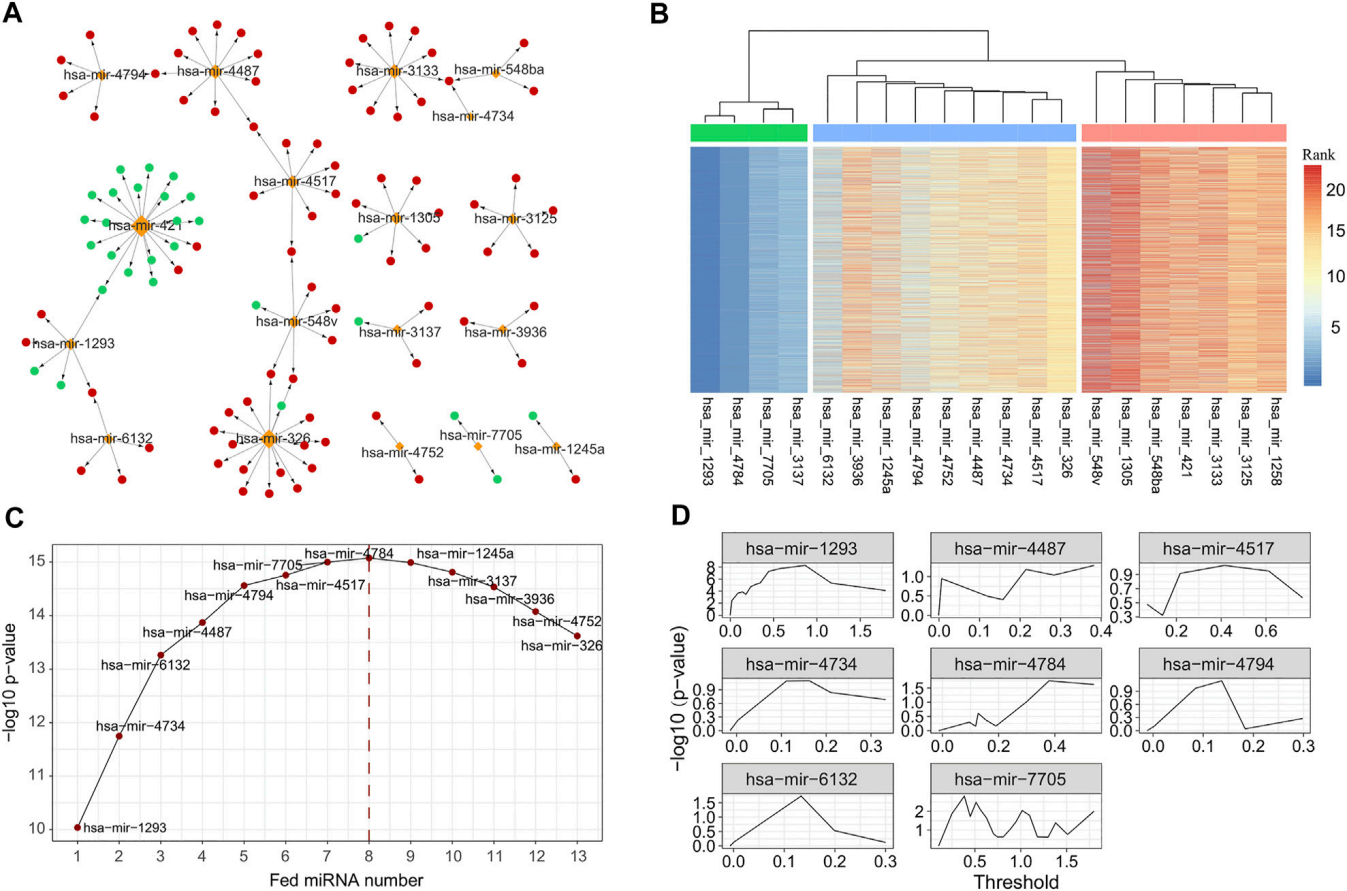
MiRNAs selected with different strategy. **(A)** The subgraph of miRNA–gene regulatory network, consisting of 26 miRNAs selected by univariate survival analysis. **(B)** Heatmap of importance rank obtained with repeatedly performed survival analysis using randomForestSRC 5,000 times. The 26 miRNAs were further clustered into three groups, and 12 miRNAs were regarded as core or important miRNAs. **(C)** Prognostic ability [measured with −log10 (*p*-value)] of miRNA combination generated by feeding the selected 12 core or important miRNAs successively. **(D)** Optimal thresholds selected for the final eight miRNA signatures. MiRNA, microRNA.

Using the negative regulation criterion and the information retrieved from three verified miRNA-target databases, we obtained 2,284 miRNA–gene pairs consisting of 228 miRNAs and 1,199 target genes. To examine the function term and effects of these miRNA regulators, we performed GO term and pathway enrichment analysis for these 1,199 target genes. The results showed that there were 924 genes functionally enriched in 700 BPs, 30 MFs, and 53 CCs with adjusted *p*-value of less than 0.05 (Supplement Table S2). Additionally, there were 163 genes enriched in 16 KEGG pathways, such as cell cycle, cellular senescence, and p53 signaling pathway (Supplement Table S2). By limiting the target genes as these functional enriched genes, we simplified the miRNA–gene regulation network consisting of 221 miRNA and 924 genes (Figure 3A).

We next performed the univariate survival analysis using the Cox proportional-hazards model with the 161 miRNA regulators. The results showed that 20 miRNAs of LUAD patients can be divided into two groups with significantly different OS (adjusted *p*-value of less than 0.05, Supplement Table S3). To further ensure the robustness of these miRNAs, we repeatedly performed survival analysis using randomForestSRC 5,000 times and measured the importance of the 21 miRNAs accordingly. With the variable importance rank matrix (see *Methods*), we clustered the 21 miRNAs into three groups using hierarchical cluster analysis (Figure 3B), and 13 miRNAs that ranked top in most of the repeats were selected for the downstream analysis.

To further select the optimal combination of the miRNA signatures, we performed multivariate survival analysis by adding the 13 miRNAs into the Cox regression model using greedy strategy (Figure 3C). By doing so, we observed that when the number of the miRNA signatures reached eight, the performance was no longer improved. Thus, we selected eight miRNAs (hsa-mir-1293, hsa-mir-4734, hsa-mir-6132, hsa-mir-4487, hsa-mir-4794, hsa-mir-4517, hsa-mir-7705, and hsa-mir-4784) as the miRNA signatures to build the risk-score prediction model.

For each of the miRNA signatures, we divided LUAD patients into two groups according to the miRNA expression with different thresholds and evaluated the discrimination validity using log-rank test and Kaplan–Meier test (Figure 3D). The optimal threshold and the β coefficients for each miRNA signature were saved for the model building (see *Methods*).

### Performance Evaluation for the Risk-Score Model

**FIGURE 4:**
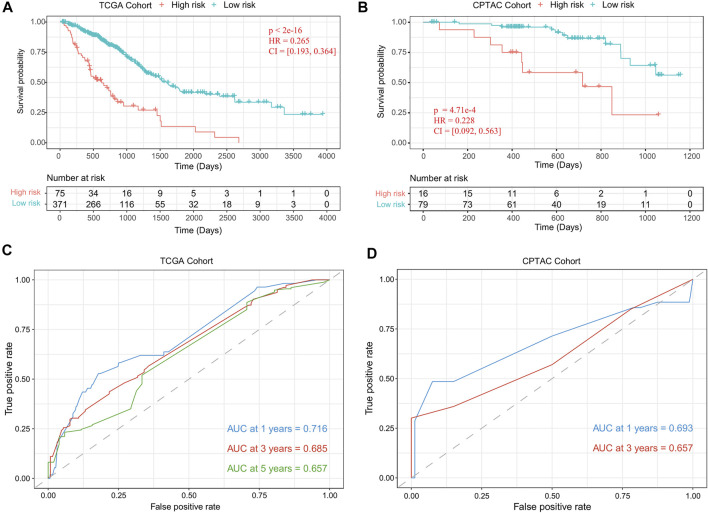
Performance evaluation of the risk-score model. **(A)** Kaplan–Meier plots of OS in TCGA-LUAD cohort when the risk-score cutoff was set as the median value (cutoff = 2.9). **(B)** Kaplan–Meier plots of OS in CPTAC-LUAD cohort when the risk-score cutoff set as 2.9. **(C)** ROC curves of risk-score model for TCGA-LUAD cohort. **(D)** ROC curves of risk-score model for the CPTAC-LUAD cohort. OS, overall survival; TCGA, The Cancer Genome Atlas; LUAD, lung adenocarcinoma; CPTAC, Clinical Proteomic Tumor Analysis Consortium; ROC, receiver operating characteristic.

Using the risk-score model, we estimated the risk score for each LUAD patient and divided the LUAD cohort into high-risk and low-risk groups by defining the cutoff as the median risk score (cutoff = 2.9). The Kaplan–Meier survival analysis results showed that the OS time was significantly different between the patients in these two groups (*p*-value = 1.43 e−18, Figure 4A). We also evaluated the performance with the independent validation dataset (CPTAC-LUAD). The risk score of the patient in the CPTAC-LUAD dataset were estimated, and then the CPTAC-LUAD patients were divided into high-risk and low-risk groups with the cutoff determined by TCGA-LUAD dataset. The Kaplan–Meier survival analysis results showed that the OS time was significantly different between the CPTAC-LUAD patients in these two groups (*p*-value = 4.71 e−4, Figure 4B).

To further assess the prognostic power of proposed method, time-dependent receiver operating characteristic (ROC) curves were used to compare the specificity and sensitivity for the predicted results of TCGA-LUAD cohort (1 year, 0.716; 3 years, 0.685; 5 years, 0.657; Figure 4C) and CPTAC-LUAD cohort (1 year, 0.693; 3 years, 0.657; Figure 4D). The ROC curves and area under the ROC curve (AUC) showed high consistency of this risk-score model.

### The Prognostic Ability of the Risk-Score Model Within Different Clinical Groups

**FIGURE 5:**
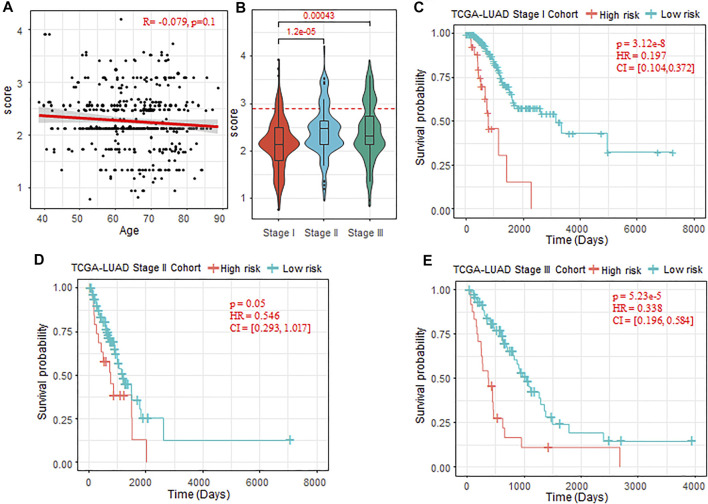
Prognostic ability of the risk-score model with different clinical factors. **(A)** Correlation between the patient age and risk score predicted. **(B)** Comparison of risk score of patients in Stage I, Stage II, and Stage III. The Wilcoxon rank sum test was used. **(C–E)** Kaplan–Meier plots of OS in Stages I–III of TCGA-LUAD cohort when the risk-score cutoff set as 2.9. OS, overall survival; TCGA, The Cancer Genome Atlas; LUAD, lung adenocarcinoma.

To further validate the prognostic ability of the risk-score model, we test the enrichment of low- and high-risk patients in the groups divided by different clinical indicators, such as age, gender, and clinical stages (Stages I–IV). We found that there was no significant difference of the risk score between the male and female patients (*p*-value = 0.133), and the risk score also did not show significant correlation with the patient age (R = −0.079, *p*-value = 0.1, Figure 5A). For the clinical stages, we found that the risk score of patients in Stage II and Stage III were significantly higher than that of patients in Stage I (Stage II: *p*-value = 1.2 e−5, Stage III: *p*-value = 4.3 e−4, Figure 5B). The low-risk patients were significantly enriched in early stage (Wilcoxon rank sum test *p*-value < 2.2 e−16). The clinical staging system is the most acknowledged clinicopathological factor for prognostication and therapy determination of LUAD, which are limited because the prognoses within the same clinical stage vary widely (Mlecnik et al., 2011). To further investigate the potentiality of the risk-score model, we tested the difference of OS between the low- and high-risk patients within the same clinical stage. The results showed that, for Stage I, Stage II, and Stage III, OS time was significantly shorter in the high-risk cohort compared with the low-risk cohort (Stage I, *p*-value = 3.12 e–8; Stage II, *p*-value = 0.05; Stage III, *p*-value = 5.23 e–5; Figures 5C–E).

### Treatment Response for the Groups Divided by the Risk-Score Model

**FIGURE 6:**
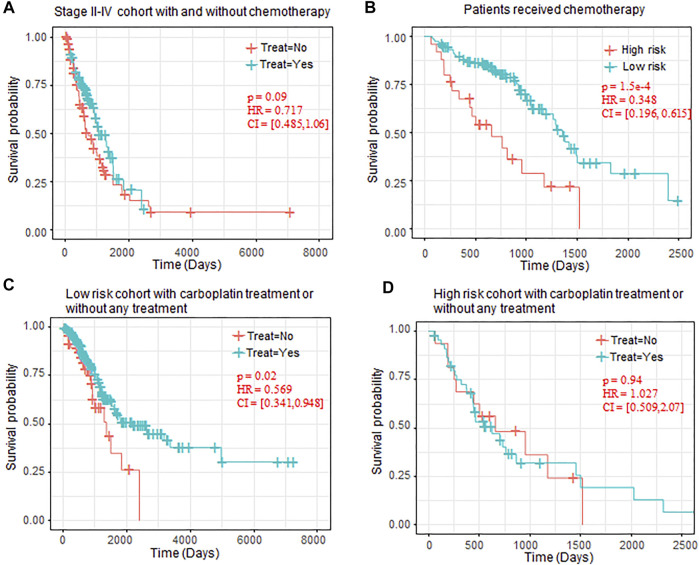
OS comparison between patients with chemotherapy. **(A)** Kaplan–Meier plots of OS in Stage II–IV patients who received chemotherapy or not. **(B)** Kaplan–Meier plots of OS in high-risk and low-risk patients who received chemotherapy. **(C)** Kaplan–Meier plots of low-risk patients who received carboplatin or without any chemotherapy. **(D)** Kaplan–Meier plots of high-risk patients who received carboplatin or without any chemotherapy. OS, overall survival.

To further evaluate the clinical benefit of the risk-score model, we extracted the treatment information for the LUAD patients, and 155 patients received different types treatment and 297 patients without any treatment information. Patients who received more than two types of therapy (e.g., patients received both chemotherapy and immunotherapy) were excluded for the follow-up analysis. As the patients who received chemotherapy were enriched in Stage II–Stage IV (Fisher’s exact test *p*-value = 2.87 e–24), we test the effectiveness of the chemotherapy on the patients in Stage II–Stage IV. The results showed that chemotherapy can improve prognosis to some extent (*p*-value = 0.09, Figure 6A).

We also observed that, in all the patients who received chemotherapy, the patients regarded as low-risk also benefited more from the chemotherapy than the high-risk chemotherapy (*p*-value = 1.5 e–4, Figure 6B). In chemotherapy drugs specifically, we also observed that carboplatin can significantly prolong the OS of low-risk patients (*p*-value = 0.02, Figure 5C), but it has no benefit in the high-risk patients (*p*-value = 0.94, Figure 5D).

## Methods

### Data Preprocessing

The quantile normalization procedure is applied to the gene and miRNA expression separately and filter out the genes and miRNAs with the expression value 0 across more than 90% of the samples. We also applied the ComBat (Leek et al., 2012) to remove the batch effect between the data in TCGA dataset and CPTAC dataset. The DESeq2 (Love et al., 2014) was used to perform the differential expression analysis between the tumor and normal samples using the raw count data. Genes with Benjamini and Hochberg adjusted *p*-value of less than 1 e–3 and fold change larger than 2 were regarded as significantly DEGs.

### Building the MicroRNA–Messenger RNA Negative Regulation Pairs

To obtain the relationship between miRNA and their target gene (mRNAs), we extracted the regulator factor miRNA of DEGs from three verified miRNA–target databases (miRecords (Xiao et al., 2009), miRTarBase (Huang et al., 2020b), and TarBase (Karagkouni et al., 2018)) using the “multiMiR” R package (Ru et al., 2014). These regulatory relationships were further refined based on the negative regulated relationship that one miRNA and its target genes were negatively related. Spearman’s correlation test was applied to each miRNA–gene pair among 504 TP samples with both miRNA expression value and mRNA expression value available, and only the pairs with negative correlation coefficient and adjusted *p*-value < 0.01 remained.

### MicroRNA Signature Selection

The procedure takes four steps to accomplish the miRNA signature selection. We first performed the functional enrichment analysis for the DEGs using the R/Biconductor package “clusterProfiler” (Yu et al., 2012), and functional terms with adjusted *p*-value of less than 0.05 were regarded as significantly enriched. We retained the miRNAs targeting the genes enriched in any functional terms. Next, we performed OS analysis for each of the remaining miRNAs, and the miRNAs with log-rank *p*-value of less than 0.05 remained. To further refine the miRNA signatures, we evaluated the extent to which each miRNA contributes to predicting survival using the metric of variable importance using the vimp function from the R package “randomForestSRC” (Ishwaran et al., 2020). We calculated variable importance using random permutation of the variable approach. To ensure robustness, we repeated this step 5,000 times, and a rank matrix for the miRNAs was obtained based on the calculated variable importance. Using the rank matrix, we divided these miRNAs into three groups (including important miRNAs, secondary miRNA, and meaningless miRNAs) using R function hclust with the default parameters. The miRNAs regarded as important or secondary were selected as candidate miRNA signatures and ranked according to the median of the 5,000 ranks of the miRNA. Finally, we performed the multivariate survival analysis using the Cox regression model by feeding the candidate miRNA signatures in sequence. The miRNAs that reduced the prognostic ability of the model were excluded. Ultimately, the rest of the miRNAs were regarded as the signatures.

### Building Risk-Score Estimator

For each miRNA signature, we calculated the optimal threshold that can divide the patients into the high-risk or low-risk group with the most significant OS time difference, and the beta (β) coefficient for each miRNA signature was also calculated with the optimal threshold. The risk score of a patient can be defined as follows:

$$\mathrm{Risk score}=\;\underset{i}{\sum }s_{i}$$

and $s_{i}$ represents the risk score for a certain miRNA i , which was calculated as follows:

si={|βi|, if βi<0 and miRNA expression lower than the related optimal threshold β, if βi>0 and miRNA expression higher than the related optimal threshold0, else

### Statistical Analysis

Time-dependent ROC curve and AUC were generated with R package “timeROC” (Blanche, 2015). Survival analysis and univariate and multivariate Cox regression analyses were performed with R package “survival” (Therneau and Lumley, 2010). The Kaplan–Meier curves were plot with R package “survminer” (Kassambara et al., 2017). Heatmap was drawn with R package “pheatmap” (Kolde and Kolde, 2015). The *p*-values of each variable were corrected using the Benjamini and Hochberg (BH) method (Benjamini and Hochberg, 1995).

## Discussion

In this study, we have identified eight miRNA signatures associated with the OS of LUAD using both the miRNA expression and gene expression profiles obtained from TCGA-LUAD dataset. With these miRNA signatures, we built a novel risk-score model using both the optimal cutoff and corresponding beta coefficients; otherwise, the miRNA expression is used directly. This model divides LUAD patients into two groups (high-risk and low-risk) with significantly different OS times. The performance was proved to be consistent in both the training set (TCGA-LUAD) and independent validation set (CPTAC-LUAD).

Through consulting literature materials, we found that all the eight miRNAs were reported to be associated with various types of cancer, including lung cancer. Additionally, personalized cancer medicine is a clinical approach that strives to customize therapies based upon the genetic profiles of individual patient tumors. Our results further proved that stratification of LUAD patients is also important to the treatment and response to therapy. However, we also noted that the clinical information, such as treatment response, in TCGA database is mainly rough, and the results in this study need further investigation in the future.

Most importantly, as built based on the optimal threshold and corresponding beta coefficients, the proposed risk-score model was fit for different types of data, including both qualitative and quantitative. This risk-score model provided a new insight into the multi-omics data integration for prognosis.

## Data Availability

The original contributions presented in the study are included in the article/Supplementary Material. Further inquiries can be directed to the corresponding author.
